# Supplementary material for: Suspicions of two bridgehead invasions of Xylella fastidiosa subsp. multiplex in France
Source: Commun Biol. 2023 Jan 27;6:103. doi: 10.1038/s42003-023-04499-6 (PMC9883466; doi:10.1038/s42003-023-04499-6)
Supplement: Supplementary file 12 — Reporting Summary [file 42003_2023_4499_MOESM12_ESM.pdf]

nature portfolio

Corresponding author(s): Marie-Agnès JACQUES

Last updated by author(s): Dec 21, 2022

Reporting Summary

Nature Portfolio wishes to improve the reproducibility of the work that we publish. This form provides structure for consistency and transparency in reporting. For further information on Nature Portfolio policies, see our Editorial Policies and the Editorial Policy Checklist.

Statistics

For all statistical analyses, confirm that the following items are present in the figure legend, table legend, main text, or Methods section.

n/a	Confirmed	
☐	☒	The exact sample size (n) for each experimental group/condition, given as a discrete number and unit of measurement
☐	☒	A statement on whether measurements were taken from distinct samples or whether the same sample was measured repeatedly
☒	☐	The statistical test(s) used AND whether they are one- or two-sided<br>*Only common tests should be described solely by name; describe more complex techniques in the Methods section.*
☒	☐	A description of all covariates tested
☒	☐	A description of any assumptions or corrections, such as tests of normality and adjustment for multiple comparisons
☐	☒	A full description of the statistical parameters including central tendency (e.g. means) or other basic estimates (e.g. regression coefficient) AND variation (e.g. standard deviation) or associated estimates of uncertainty (e.g. confidence intervals)
☐	☒	For null hypothesis testing, the test statistic (e.g. F, t, r) with confidence intervals, effect sizes, degrees of freedom and P value noted<br>*Give P values as exact values whenever suitable.*
☐	☒	For Bayesian analysis, information on the choice of priors and Markov chain Monte Carlo settings
☒	☐	For hierarchical and complex designs, identification of the appropriate level for tests and full reporting of outcomes
☒	☐	Estimates of effect sizes (e.g. Cohen's d, Pearson's r), indicating how they were calculated

Our web collection on statistics for biologists contains articles on many of the points above.

Software and code

Policy information about availability of computer code

Data collection	NA

Data analysis	Tandem Repeat Finder v4.09<br>Geneious v9.1.8<br>PHYLOViZ v2.0<br>STRUCTURE v2.3.4<br>BioNumerics v7.6<br>Fstat v2.9.4<br>GenAlEx v5.5.1<br>Arlequin v3.5.2.2<br>DIYABC software v2.1<br>SOAPdenovo v2.04<br>SOAPGapCloser v1.12<br>Velvet v1.2.02<br>Harvest suite: Parsnp tool v1.2, gr tool v1.3<br>RAxML v8.2.4<br>BEAST v2.6.1<br>Tracer v1.7.1<br>LogCombiner v2.6.1<br>Tree-Annotator v2.6<br>R packages: abcrf, adegenet, ape, coda, gplots, maptools

For manuscripts utilizing custom algorithms or software that are central to the research but not yet described in published literature, software must be made available to editors and reviewers. We strongly encourage code deposition in a community repository (e.g. GitHub). See the Nature Portfolio guidelines for submitting code & software for further information.

Data

Policy information about availability of data

All manuscripts must include a data availability statement. This statement should provide the following information, where applicable:

- Accession codes, unique identifiers, or web links for publicly available datasets
- A description of any restrictions on data availability
- For clinical datasets or third party data, please ensure that the statement adheres to our policy

Strains of X. fastidiosa were deposited at the CIRM-CFBP (International Centre of Microbial Resource (CIRM) - French Collection for Plant-associated Bacteria. INRAE. https://doi.org/10.15454/E8XX-4Z18).
Genome sequences were deposited at NCBI under the accession numbers listed in Table S1.
All data are available in the main text or the supplementary materials.

Field-specific reporting

Please select the one below that is the best fit for your research. If you are not sure, read the appropriate sections before making your selection.

☐ Life sciences ☐ Behavioural & social sciences ☒ Ecological, evolutionary & environmental sciences

For a reference copy of the document with all sections, see nature.com/documents/nr-reporting-summary-flat.pdf

Ecological, evolutionary & environmental sciences study design

All studies must disclose on these points even when the disclosure is negative.

Study description	This study describes the study of the diversity of the Xylella fastidiosa bacterium in France, as well as the research of its routes of introduction in our country and their dating.
Research sample	Samples are composed of Xylella fastidiosa French strains isolated from infected plants and infected plants sampled bewteen 2015 and 2018
Sampling strategy	The samples used in this study were sampled in the framework of the national official surveillance strategy for X. fastidiosa and other dedicated sampling campaigns between 2015 and 2018 based on visual symptoms. We were allowed to study and used these official samples in our study
Data collection	The samples used in this study were sampled in the framework of the national official surveillance strategy for X. fastidiosa and other dedicated sampling campaigns between 2015 and 2018
Timing and spatial scale	Samples were sampled between 2015 and 2018
Data exclusions	No data exclusion
Reproducibility	For MLVA analyses, the VNTR-13 profiles of 13 strains and the plant from which they were isolated were compared and the results were exactly the same.<br>For the data analyses, the tests were always performed multiple times.

Randomization	NA
Blinding	Samples were always analyzed using a code number and linked subsequently to temporal and geographical data.

Did the study involve field work? ☒ Yes ☐ No

Field work, collection and transport

Field conditions	NA
Location	French strains and infected samples were collected in Corsica or PACA regions of France
Access & import/export	These infected samples were collected in Corsica and in PACA regions of France between 2015 and 2018 in the framework of the national official surveillance strategy for X. fastidiosa and other dedicated sampling campaigns
Disturbance	NA

Reporting for specific materials, systems and methods

We require information from authors about some types of materials, experimental systems and methods used in many studies. Here, indicate whether each material, system or method listed is relevant to your study. If you are not sure if a list item applies to your research, read the appropriate section before selecting a response.

Materials & experimental systems

n/a	Involved in the study	
☒	☐	Antibodies
☒	☐	Eukaryotic cell lines
☒	☐	Palaeontology and archaeology
☒	☐	Animals and other organisms
☒	☐	Human research participants
☒	☐	Clinical data
☒	☐	Dual use research of concern

Methods

n/a	Involved in the study	
☒	☐	ChIP-seq
☒	☐	Flow cytometry
☒	☐	MRI-based neuroimaging
